# Epilepsy in the mTORopathies: opportunities for precision medicine

**DOI:** 10.1093/braincomms/fcab222

**Published:** 2021-09-25

**Authors:** Patrick B Moloney, Gianpiero L Cavalleri, Norman Delanty

**Affiliations:** FutureNeuro, the Science Foundation Ireland Research Centre for Chronic and Rare Neurological Diseases, Royal College of Surgeons in Ireland, Dublin, D02 VN51, Ireland; Blackrock Clinic, Dublin, A94 E4X7, Ireland; Department of Neurology, Beaumont Hospital, Dublin, D09 V2N0, Ireland; School of Pharmacy and Biomolecular Sciences, Royal College of Surgeons in Ireland, Dublin, D02 VN51, Ireland; FutureNeuro, the Science Foundation Ireland Research Centre for Chronic and Rare Neurological Diseases, Royal College of Surgeons in Ireland, Dublin, D02 VN51, Ireland; School of Pharmacy and Biomolecular Sciences, Royal College of Surgeons in Ireland, Dublin, D02 VN51, Ireland; FutureNeuro, the Science Foundation Ireland Research Centre for Chronic and Rare Neurological Diseases, Royal College of Surgeons in Ireland, Dublin, D02 VN51, Ireland; Department of Neurology, Beaumont Hospital, Dublin, D09 V2N0, Ireland; School of Pharmacy and Biomolecular Sciences, Royal College of Surgeons in Ireland, Dublin, D02 VN51, Ireland

**Keywords:** the mTORopathies, tuberous sclerosis complex, GATOR1-related epilepsies, focal cortical dysplasia type II, everolimus

## Abstract

The mechanistic target of rapamycin signalling pathway serves as a ubiquitous regulator of cell metabolism, growth, proliferation and survival. The main cellular activity of the mechanistic target of rapamycin cascade funnels through mechanistic target of rapamycin complex 1, which is inhibited by rapamycin, a macrolide compound produced by the bacterium *Streptomyces hygroscopicus*. Pathogenic variants in genes encoding upstream regulators of mechanistic target of rapamycin complex 1 cause epilepsies and neurodevelopmental disorders. Tuberous sclerosis complex is a multisystem disorder caused by mutations in mechanistic target of rapamycin regulators *TSC1* or *TSC2,* with prominent neurological manifestations including epilepsy, focal cortical dysplasia and neuropsychiatric disorders. Focal cortical dysplasia type II results from somatic brain mutations in mechanistic target of rapamycin pathway activators *MTOR, AKT3, PIK3CA and RHEB* and is a major cause of drug-resistant epilepsy. *DEPDC5*, *NPRL2* and *NPRL3* code for subunits of the GTPase-activating protein (GAP) activity towards Rags 1 complex (GATOR1), the principal amino acid-sensing regulator of mechanistic target of rapamycin complex 1. Germline pathogenic variants in GATOR1 genes cause non-lesional focal epilepsies and epilepsies associated with malformations of cortical development. Collectively, the mTORopathies are characterized by excessive mechanistic target of rapamycin pathway activation and drug-resistant epilepsy. In the first large-scale precision medicine trial in a genetically mediated epilepsy, everolimus (a synthetic analogue of rapamycin) was effective at reducing seizure frequency in people with tuberous sclerosis complex. Rapamycin reduced seizures in rodent models of *DEPDC5*-related epilepsy and focal cortical dysplasia type II. This review outlines a personalized medicine approach to the management of epilepsies in the mTORopathies. We advocate for early diagnostic sequencing of mechanistic target of rapamycin pathway genes in drug-resistant epilepsy, as identification of a pathogenic variant may point to an occult dysplasia in apparently non-lesional epilepsy or may uncover important prognostic information including, an increased risk of sudden unexpected death in epilepsy in the GATORopathies or favourable epilepsy surgery outcomes in focal cortical dysplasia type II due to somatic brain mutations. Lastly, we discuss the potential therapeutic application of mechanistic target of rapamycin inhibitors for drug-resistant seizures in GATOR1-related epilepsies and focal cortical dysplasia type II.

## Teaser

Moloney et al. outline the spectrum of epilepsies caused by pathogenic variation in genes encoding for regulators of the mechanistic target of rapamycin signalling pathway. Epileptogenesis in the mTORopathies results from excessive activation of the pathway, presenting a therapeutic target for seizures and other manifestations in these disorders.

## Graphical Abstract

**Graphical Abstract:**
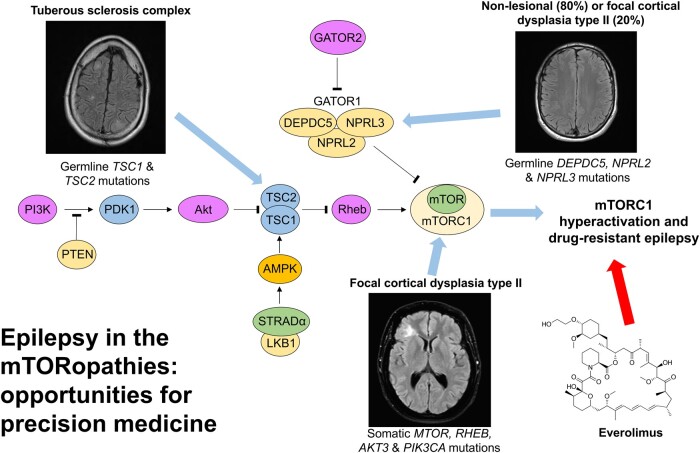

## Introduction

Advances in next-generation sequencing technology and associated data analytics have accelerated genetic discovery in the epilepsies over the past two decades. Gene discovery in the developmental and epileptic encephalopathies (DEEs) has been particularly fruitful, with a specific genetic cause identified in up to 40% of cases.^1^^,^^2^ Evidence is now emerging for a significant monogenic contribution to the focal epilepsies.^3^^,^^4^ Genetic data increasingly inform clinical decision-making. Our enhanced understanding of the molecular mechanisms underpinning some monogenic epilepsies has prompted repurposing of existing drugs that target specific genetic mechanisms and development of novel precision therapies. Precision medicine aims to customise treatment to the personalized characteristics of individuals, including their genetic data.^5^ The majority of available treatments for epilepsy are imprecise symptomatic therapies, lacking true disease-modifying properties.

Epilepsy is fundamentally a disorder of neural networks, with seizures being the most visible manifestation and the target of the currently available symptomatic therapies. Disrupted brain networks may also account for the neuropsychiatric and neurodevelopmental comorbidities commonly seen in the genetic epilepsies. A broad range of disease mechanisms underlies the monogenic epilepsies including ion-channel dysfunction, dysregulation of synaptic processes, mechanistic target of rapamycin (mTOR) pathway hyperactivation and impaired chromatin remodelling and transcription regulation.^5^^,^^6^ For the purpose of this review, we will focus on the genetic epilepsies that result from dysregulation of the mTOR cascade, collectively referred to as mTORopathies.

The mTOR signalling pathway serves as a ubiquitous regulator of cell metabolism, growth, proliferation and survival. Pathogenic variation in genes encoding regulators of the mTOR cascade cause epilepsies, malformations of cortical development (MCD) and neurodevelopmental disorders.^7^ The neurological manifestations of tuberous sclerosis complex (TSC) include drug-resistant epilepsy (DRE), focal cortical dysplasia (FCD) and neuropsychiatric disorders.^8^ Brain somatic mutations in mTOR pathway genes are a common cause of FCD and hemimegalencephaly (HME).^9^^,^^10^ Pathogenic variants in genes encoding the GTPase-activating protein (GAP) activity towards Rags 1 complex (GATOR1) cause non-lesional focal epilepsies and FCD-related epilepsies.^11^ Everolimus, an mTOR inhibitor, was effective at reducing focal seizures in TSC, in the first large-scale precision medicine trial in genetically mediated epilepsy.^12^ The anti-seizure effects of mTOR inhibition may also be applicable to other mTORopathies, as excessive activation of the mTOR pathway appears to be an essential pathomechanism for the development of epilepsy in all of these disorders.

In this review, we delineate the spectrum of epilepsies and MCD in the mTORopathies, as well as outlining the influence of mTOR pathway hyperactivation in the pathogenesis of these disorders. We evaluate the use mTOR inhibitors as treatments for the various manifestations of TSC, including seizures and postulate potential applications in other mTORopathies. Lastly, we identify precision medicine opportunities in the epilepsies associated with TSC, the GATOR1-related disorders and FCD type II.

## The mTOR cascade

**Figure 1:**
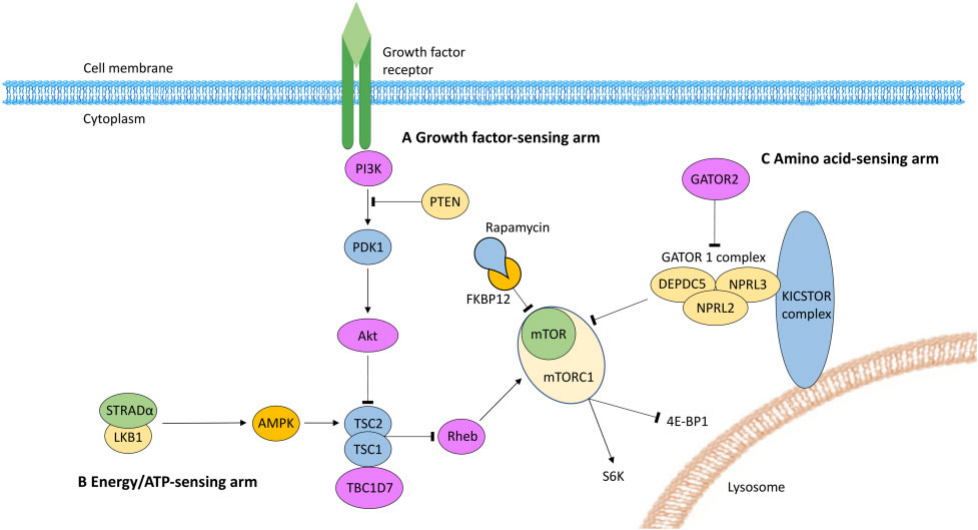
**The mTOR cascade and its regulators. Mechanistic target of rapamycin (mTOR) combines with protein binding partners to form mTOR complex 1 (mTORC1).** The downstream substrates of mTORC1 signalling include ribosomal S6 kinase (S6K) and eukaryotic initiation factor 4E-binding protein-1 (4E-BP1). Rapamycin inhibits mTORC1 signalling by binding with FK506 binding protein 1 A 12 kDa (FKBP12). Three pathways converge to regulate mTORC1 signalling. Hamartin (TSC1), tuberin (TSC2) and TBC1 domain family member 7 (TBC1D7) form a protein complex that indirectly inhibits mTORC1 signalling via Ras homologue enriched in brain (Rheb). (**A**) Growth factors stimulate phosphoinositide 3-kinase (PI3K) to trigger phosphoinositide-dependent kinase 1 (PDK1) to phosphorylate and activate Akt. TSC2 is repressed by Akt activation, which has a disinhibitory effect on mTORC1 signalling. Phosphatase and tensin homologue (PTEN) is a negative regulator of the PI3K-Akt pathway. (**B**) The energy-sensing arm is regulated by the STE20-related kinase adaptor alpha (STRADα) and liver kinase B (LKB) complex. In response to depleted ATP, the STRADα/LKB complex inhibits mTORC1 signalling by activating TSC2 via phosphorylation of adenosine monophosphate-activated kinase (AMPK). (**C**) The amino acid-sensing pathway is regulated by GTPase-activating protein (GAP) activity towards Rags 1 complex (GATOR1). GATOR1 is composed of three subunits: Dishevelled, Egl-10, and Pleckstrin domain-containing protein 5 (DEPDC5); nitrogen permease regulator-like 2 and 3 (NPRL2, NPRL3). The GATOR2 complex inhibits GATOR1 in response to increasing amino acid levels, resulting in mTORC1 disinhibition, facilitating pathways for cell growth. When amino acid levels are low GATOR1 directly inhibits mTORC1 activity. The KICSTOR (KPTN, ITFG2, C12orf66 and SZT2-containing regulator of mTORC1) complex scaffolds GATOR1 to the lysosomal surface (adapted from Peter B. Crino’s review^7^ with permission from Springer Nature).

The serine/threonine protein kinase mTOR is ubiquitously expressed, with particularly high levels in the brain. mTOR combines with binding partners to form mTOR complex 1 (mTORC1) and mTOR complex 2 (mTORC2). mTORC1 is a central signalling node, receiving inputs from upstream regulatory proteins that are influenced by growth factors (for example, insulin), ATP concentrations and nutrients (for example, amino acids). When activated, mTORC1 promotes cell growth and survival via regulation of messenger RNA translation, nucleotide biosynthesis and autophagy.^7^^,^^13^^,^^14^ Downstream substrates of mTORC1 signalling that modulate these pivotal cellular processes include ribosome S6 kinase (S6K) and eukaryotic initiation factor 4E-binding protein.^14^ In the brain, mTORC1 has important functions related to synaptic transmission and plasticity, neural network activity and neurogenesis.^7^^,^^15^ Rapamycin (also known as sirolimus) is produced by the bacterium *Streptomyces hygroscopius* and is an inhibitor of mTORC1 signalling. Rapamycin inhibits mTORC1 activity by forming a complex with FK506 binding protein 1 A 12 kDa (FKBP12). The FKBP12-rapamycin binding complex interacts with mTOR and inhibits mTORC1 by an allosteric mechanism.^7^ mTORC2 is primarily involved in cytoskeletal integrity and cell migration, and is insensitive to rapamycin inhibition.^16^ The activity of mTORC1 is mainly regulated by three converging signalling pathways: the growth factor pathway; the energy/ATP-sensing pathway; and the amino acid-sensing pathway.^7^ The mTOR cascade has been described in detail elsewhere,^7^^,^^14^^,^^17^ and is summarized in Fig. 1.

The amino acid-sensing arm of the mTOR cascade has garnered recent attention due to its role in a number of genetic focal epilepsies and DEEs. GATOR1 is composed of three subunits: Dishevelled, Egl-10 and Pleckstrin domain-containing protein 5 (DEPDC5); nitrogen permease regulator-like 2 (NPRL2); and nitrogen permease regulator-like 3 (NPRL3). The GATOR2 complex inhibits GATOR1 in response to increasing amino acid levels, resulting in mTORC1 disinhibition, facilitating pathways for cell growth. When amino acid levels are low, GATOR1 directly inhibits mTORC1 activity.^13^ Kaptin (KPTN), integrin alpha FG-GAP repeat containing 2 (ITFG2), chromosome 12 open reading frame 66 (C12orf66) and seizure threshold 2 (SZT2) form the KICSTOR complex (KPTN, ITFG2, C12orf66 and SZT2-containing regulator of mTORC1). The KICSTOR complex scaffolds GATOR1 to the lysosomal surface.^18^

## The molecular and clinical spectrum of mTORopathies

Both germline and somatic mutations in genes encoding for different components of mTOR signalling cause epilepsies, MCD and neurodevelopmental disorders. Loss-of-function (LoF) mutations in pathway inhibitors (*TSC1, TSC2, DEPDC5, NPRL3, NPRL2, PTEN, STRADA*) or gain-of-function (GoF) mutations in pathway activators (*PI3KCA, AKT3, RHEB, MTOR*) culminate in hyperactivation of mTORC1.^19^ To date, pathogenic variants in 16 distinct genes encoding mTOR pathway proteins have been detected in individuals with epilepsy and/or neurodevelopmental disorders (Table 1). The mTORopathies comprise of a spectrum of MCD that range from whole brain (megalencephaly) and hemispheric (HME) abnormalities, to focal abnormalities such as FCD and bottom-of-sulcus dysplasia (BOSD), to ‘normal’ appearing brain on high resolution MRI. GATOR1 pathogenic variants most frequently cause non-lesional focal epilepsies.^11^ Epilepsy may result from subtle FCD that is not detectable by conventional imaging techniques in an unknown proportion of people with GATOR1 mutations and normal high-resolution MRI.^20^ mTOR-related MCD is often associated with intellectual disability and/or autism spectrum disorders (ASD).

**Table 1:** mTOR pathway proteins (and their encoding genes) associated with epilepsy and malformations of cortical development

mTOR pathway protein(s)	Normal effect on mTORC1 signalling	Mutation	Epilepsy syndrome	Malformation of cortical development
TSC1 and TSC2	Inhibition (TSC protein complex)	Germline *TSC1* or *TSC2*	TSC; focal epilepsy with structural cause	Tuber; FCD type II^9^; HME^30^^,^^141^
		Somatic *TSC1* or *TSC2*	TSC^70^; focal epilepsy with structural cause	Tuber; FCD type II^9^^,^^10^^,^^30^^,^^142^; HME^30^
TBC1D7	Inhibition (TSC protein complex)	Germline *TBC1D7* (AR)	No reports of seizures but epileptiform discharges on EEG^143^	Megalencephaly^144^
DEPDC5, NPRL2 and NPRL3	Inhibition (GATOR1 complex)	Germline *DEPDC5, NPRL2 and NPRL3*	Focal epilepsy with no structural cause on MRI brain; focal epilepsy with structural cause	FCD type II^9–11^^,^^30^^,^^31^^,^^36^^,^^61–63^; BOSD^26^^,^^61^^,^^121^; HME^31^^,^^122^
				FCD type I^11^^,^^36^^,^^63^; subcortical band heterotopia^121^; polymicrogyria^4^; pachygyria^145^
		Somatic *DEPDC5*	Focal epilepsy with structural cause	Second hit somatic mutations in FCD type I^36^ and type II^9^^,^^10^^,^^34^^,^^35^
mTOR	Activation (component of mTORC1)	Germline *MTOR*	Focal epilepsy with no structural cause on MRI brain^146^; Smith–Kingsmore syndrome (seizures in 73.9% of cases)^22^	HME^22^; megalencephaly^22^
		Somatic *MTOR*	Focal epilepsy with structural cause	FCD type II^9^^,^^10^^,^^28^^,^^30^^,^^31^^,^^95^^,^^146^; BOSD^26^; HME^9^^,^^10^^,^^29–31^; megalencephaly^22^; polymicrogyria^59^
			Smith–Kingsmore syndrome^22^	
Akt	Activation (component PI3K-Akt pathway)	Germline *AKT3*	MPPH syndrome (seizures in 47% of cases)^147^	Polymicrogyria^23^^,^^24^; megalencephaly^23^^,^^24^
		Somatic *AKT1*	Focal epilepsy with structural cause	HME^30^^,^^148^; megalencephaly^148^
			Proteus syndrome (rare seizures)^148^	
		Somatic *AKT3*	Focal epilepsy with structural cause	FCD type II^9^; HME^9^^,^^10^^,^^27^^,^^30^^,^^31^
PI3K	Activation (component PI3K-Akt pathway)	Germline *PIK3CA*	MCAP syndrome (seizures in 20% of cases)^147^	Megalencephaly^149^; polymicrogyria^149^
		Germline *PIK3R2*	MPPH syndrome (seizures in 47% of cases)^23^^,^^147^; focal epilepsy with structural cause	Megalencephaly^150^; polymicrogyria^150^
		Somatic *PIK3CA*	Focal epilepsy with structural cause	FCD type II^27^; HME^9^^,^^10^^,^^27^^,^^30^^,^^31^; megalencephaly^27^; polymicrogyria^23^
			MCAP syndrome (seizures in 20% of cases)^23^^,^^147^; MPPH syndrome (seizures in 47% of cases)^23^^,^^147^	
		Somatic *PIK3R2*	Focal epilepsy with structural cause	Megalencephaly^150^; polymicrogyria^150^
STRADα	Inhibition	Germline *STRADA* (AR)	PMSE syndrome (infantile-onset DEE)^21^	Megalencephaly^21^; subependymal dysplasia^21^
PTEN	Inhibition of PI3K-Akt pathway	Germline *PTEN*	Focal epilepsy with structural cause Bannayan-Riley-Ruvalcaba syndrome (seizures in 25% of cases)^151^; Cowden syndrome (rare seizures)^152^; ASD and macrocephaly syndrome (rare seizures)^153^	FCD^154^; HME^27^; megalencephaly^153^; polymicrogyria^155^; subependymal heterotopia^155^
		Somatic *PTEN*	Focal epilepsy with structural cause	Two distinct somatic *PTEN* mutations in a case of HME^156^
Rheb	Activation	Somatic *RHEB*	Focal epilepsy with structural cause	FCD type II^9^^,^^60^; HME^9^
SZT2	Inhibition (KICSTOR complex)	*SZT2 g*ermline (AR)	Infantile-onset DEE^157^^,^^158^	Megalencephaly^157^^,^^158^
Kaptin	Inhibition (KICSTOR complex)	*KPTN g*ermline (AR)	Infantile-onset DEE^159^^,^^160^	Megalencephaly^159^^,^^160^

AR, autosomal recessive; ASD, autism spectrum disorder; BOSD, bottom-of-sulcus dysplasia; DEE, developmental and epileptic encephalopathy; FCD, focal cortical dysplasia; HME, hemimegalencephaly; MCAP, megalencephaly capillary malformation-polymicrogyria; MPPH, megalencephaly polydactyly polymicrogyria-hydrocephalus; PMSE, polyhydramnios, hydrocephalus and symptomatic epilepsy; TSC, tuberous sclerosis complex.

Some mTORopathies are multisystem disorders (for example, TSC), whilst others have a ‘brain only’ phenotype (for example, *DEPDC5*-related epilepsies) (Box 1). The mechanisms that dictate the pattern of organ involvement in the various mTORopathies are poorly understood, but may relate to organ-specific gene expression, the normal function of the faulty protein within the mTOR cascade, and the timing of mutagenesis. In addition to the more prevalent mTORopathies, like TSC and the GATOR1-related epilepsies, there are a number of very rare multisystem disorders including polyhydramnios, megalencephaly and symptomatic epilepsy (PMSE), Smith–Kingsmore syndrome, megalencephaly polydactyly polymicrogyria-hydrocephalus syndrome (MPPH) and megalencephaly capillary malformation-polymicrogyria syndrome (MCAP).^21–24^

**Box 1:** ‘Brain only’ versus multisystem mTORopathies

**‘Brain only’ mTORopathies** GATOR1-related focal epilepsies (germline *DEPDC5, NPLR2 and NPRL3)* Focal cortical dysplasia type II (somatic *MTOR, AKT3, PIK3CA and RHEB)* Hemimegalencephaly (somatic *MTOR, AKT3, PIK3CA, RHEB* and *PTEN)* Infantile-onset developmental and epileptic encephalopathies (germline *SZT2* and *KPTN)*^a^
**Multisystem mTORopathies** Tuberous sclerosis complex (germline and somatic *TSC1 and TSC2)* Smith–Kingsmore syndrome (germline and somatic *MTOR)*^a^ Dysmorphic facial features, intellectual disability, herniae, hypomelanosis, small thorax Polyhydramnios, megalencephaly, and symptomatic epilepsy (germline *STRADA)*^a^ Polyhydramnios, facial dysmorphism, intellectual disability, skeletal deformity, cardiac anomalies Megalencephaly capillary malformation syndrome (germline and somatic *PIK3CA*)^a^ Cutaneous vascular malformations, intellectual disability, digital abnormalities, cardiac anomalies Megalencephaly Polymicrogyria-Polydactyly Hydrocephalus (germline *AKT3* and *PIK3R2*)^a^ Intellectual disability, postaxial polydactyly, facial dysmorphism Proteus syndrome (somatic *AKT1*)^a^ Patchy or segmental overgrowth and hyperplasia of multiple tissues Bannayan–Riley–Ruvalcaba syndrome (germline *PTEN*)^a^ Childhood onset, macrocephaly, lipomas, hamartomas, intellectual disability Cowden syndrome (germline *PTEN)*^a^ Adult onset, multiple hamartomas, increased cancer risk, Lhermitte-Duclos disease, macrocephaly CLOVES syndrome (somatic *PIK3CA)*^a^ Lipomatous overgrowth, lymphatic malformations, vascular malformations, skeletal anomalies TBC1D7-related macrocephaly (germline *TBC1D7)*^a^ Patellar dislocation, osteoarticular anomalies, coeliac disease, intellectual disability

a Very rare mTORopathies.

The mTORopathies share common neuropathological features including abnormal cellular morphology and enlargement (cytomegaly), disorganized cortical lamination, neuronal hyperexcitability and constitutive mTORC1 signalling.^19^ FCD type II is a major cause of childhood-onset DRE and is categorized as an mTORopathy based on its molecular and cellular traits. FCD type IIA is characterized by cortical dyslamination and dysmorphic neurons. FCD type IIB is distinguished from FCD type IIA by the presence of balloon cells. HME is at the severe end of the FCD spectrum and is characterized by enlargement of part or all of one hemisphere, often with histological features of FCD type II.^25^ BOSD is at the milder end of the spectrum, with signal change and cortical thickening at the bottom of a sulcus on MRI and histological features consistent with FCD type II.^26^ Cortical and subcortical tubers in TSC have been reclassified as FCD, as they share histopathological features with FCD type II, including disorganized lamination and dysmorphic cytomegalic neurons.^25^

Low-level mosaic somatic mutations in mTOR pathway activating genes (*MTOR, AKT3, PIK3CA, RHEB*) are a major cause of FCD type II and HME.^9^^,^^10^,^26–31^ Germline *TSC1, TSC2*, *DEPDC5, NPRL2* and* NPRL3* variants are also associated with FCD type II and HME.^8^^,^^11^ Knudson’s^32^ two-hit mechanism is proposed for FCD seen in patients with germline mTOR pathway genetic mutations. Second-hit somatic mutations have been demonstrated in surgically resected FCD (including HME and tubers) from patients with germline *TSC1, TSC2 and DEPDC5* variants.^9^^,^^10^^,^^30^^,^^33–37^

## mTOR pathway hyperactivation and epileptogenesis

The precise mechanisms by which mTORopathies cause neuronal hyperexcitability and seizures remain to be fully defined but excessive mTORC1 activation appears to be implicated in epileptogenesis by disrupting the formation of neural circuits and by altering established neural networks.^20^ The extent to which seizures are a direct consequence of mTORC1 hyperactivation rather than a corollary of network disruption due to structural cortical malformation remains unresolved. In a rodent model of biallelic *Tsc1* deletion, mice developed early severe seizures, without significant alteration of brain structure, supporting the theory that excessive mTOR activation alone is sufficient to generate seizures.^38^

Dysmorphic neurons and balloon cells are considered a neuropathological hallmark of aberrant mTOR signalling, as they consistently display enhanced mTORC1 activation and are the main carriers of somatic mutations in FCD.^9^^,^^39–41^*In vitro* electrophysiological studies of FCD tissue have shown that dysmorphic and immature neurons play an important role in the generation and propagation of epileptic discharges, while balloon cells lack epileptogenicity.^42^ Moreover, FCD type II and cortical tubers retain immature GABA signalling mechanisms, resulting in abnormal neural networks and hyperexcitable cortical foci.^43^^,^^44^ In the immature CNS, GABA acts as an excitatory neurotransmitter, in contrast to its inhibitory function in the developed CNS.^45^ Persistence of immature GABA receptors leads to the development of spontaneous pacemaker GABA receptor-mediated synaptic activity, that produces self-sustaining abnormal epileptogenic discharges.^46^

Abnormalities in dendritic spine morphology and glutamatergic synaptic transmission were observed in rodent models of *Tsc1 and Tsc2* knockout. These alterations in neuronal structure and function are likely to contribute to the pathogenesis of epilepsy in TSC.^47^ In a mouse model of *DEPDC5*-related FCD developed using *in utero* electroporation and CRISPR/Cas9 technology to recapitulate a second-hit somatic mutation in cortical pyramidal cells, *Depdc5* inactivation led to abnormal dendritic and spine shaping, increased excitatory transmission and epileptogenesis.^34^

Hyperactivity of the mTORC1 pathway has been demonstrated in: experimental animal models of mTORopathies; *in vitro* functional assessments of epilepsy-causative mTOR pathway genetic variants; and resected brain tissue from patients with FCD and HME.^13^ mTORC1 activity is assessed by immunostaining for downstream substrates of mTORC1 activation, such as phosphorylated ribosomal S6K.

## Animal models of mTORopathies

Preclinical rodent models of mTORopathies have been developed to study the effects of knockdown or conditional knockout of mTOR pathway inhibitors or overexpression of mTOR pathway activators. Conditional knockout models of *Pten, Tsc1 and Tsc2* were associated with disorganized cortical cytoarchitecture, cytomegalic neurons, mTORC1 hyperactivation and rapamycin-responsive seizures.^48–50^ In TSC models, the neurological phenotype was almost completely prevented by early treatment with rapamycin.^49^^,^^50^*Strada* knockdown in a mouse model of PMSE resulted in ventricular heterotopic neurons. Rapamycin rescued the cortical migratory defect.^21^ Murine FCD models have been developed using *in utero* electroporation to produce focal cortical expression of mutant *Mtor* and *Rheb*. Mutant mice with FCD displayed dysmorphic neurons and spontaneous seizures, both almost completely rescued by rapamycin treatment.^28^^,^^51^*Depdc5* knockdown rat models displayed cytomegalic dysmorphic neurons, hyperexcitable cortical neurons, markers of mTORC1 upregulation and lowered seizure thresholds.^52^^,^^53^ Experimental models of FCD developed using *in utero* electroporation combined with CRISPR-editing of *Depdc5* in rodent cortex resulted in enlarged neurons, hyperactivated mTORC1, clinical seizures and sudden death.^34^^,^^54^ In another *Depdc5* conditional knockout model, mice displayed increased phosphorylated S6K immunostaining, thickened cortex, cytomegalic neurons, clinical seizures and premature death. Rapamycin reduced seizure frequency and extended survival in this *Depdc5* model.^55^

## *In vitro* functional assays

*In vitro* functional assessments of mTOR pathway genetic variants previously detected in people with epilepsy demonstrated molecular evidence of mTORC1 hyperactivation. These functional assays are performed in transfected heterologous systems. Functional assessments of some *TSC1*, *TSC2, DEPDC5, NPRL2, NPRL3* and* STRADA* variants (all genes encoding negative regulators of mTORC1) were associated with increased mTORC1 activity.^21^^,^^56–58^*In vitro* studies of genes encoding pathway activators such as *AKT3*, *PIK3R2, PIK3C*A and *MTOR* also displayed increased immunostaining for downstream substrates of mTORC1.^23^^,^^28^ Importantly, a significant proportion of the GATOR1 variants studied *in vitro* (just under 70%) were not associated with increased phosphorylated S6K immunostaining.^57^^,^^58^ However, these functional assays may not reflect the *in vivo* behaviour of some GATOR1 variants and other aspects unrelated to mTORC1 signalling may produce the epilepsy phenotype.

## Resected human brain tissue

Resected FCD type II and HME specimens consistently demonstrate evidence of enhanced constitutive mTORC1 activation.^33^^,^^39–41^ Increased mTORC1 activity has been shown in FCD type II and HME specimens from patients with somatic mutations in mTOR pathway activating genes (*MTOR, PIK3CA, AKT3* and* RHEB*).^9^^,^^26–28^^,^^59^^,^^60^ Resected FCD type II and HME specimens from patients with germline GATOR1 variants (*DEPDC5, NPRL2* and* NPRL3*) have also displayed enhanced phosphorylated S6K expression.^9^^,^^26^^,^^61–63^ Importantly, FCD type II specimens display evidence of mTORC1 hyperactivation, irrespective of the presence of detectable somatic or germline variants, consistent with the hypothesis that all FCD type II are mosaic mTORopathies.^9^ A mutational gradient has been demonstrated in FCD type II specimens harbouring somatic mutations. Tissues with increased concentrations of dysplastic cells have higher rates of mosaicism. The mutational load is lower in the surrounding epileptogenic zone, with somatic mutations absent from adjacent normal tissue.^9^^,^^26^^,^^34^^,^^35^^,^^59^

## Personalized medicine in mTORopathy-related epilepsies

### Polyhydramnios, megalencephaly and symptomatic epilepsy

PMSE is a very rare autosomal recessive multisystem disorder characterized by infantile-onset DRE, severe cognitive impairment, skeletal deformity and craniofacial dysmorphism. Original descriptions of the disorder came from the Old Order Mennonite population in the USA, with all cases caused by a homozygous truncating deletion of exons 9 to 13 in the *STRADA* gene. In total, 16 patients carrying the homozygous *STRADA* deletion of exons 9 to 13 have been reported.^21^ In addition, six phenotypically similar cases with novel *STRADA* variants have been described.^64–67^ STE20-related kinase adaptor alpha (STRADα) acts as an mTOR repressor in the ATP-sensing arm of the pathway. Data from *Strada* knockdown mouse models, *in vitro* functional studies of STRADα depletion and a post-mortem PMSE brain specimen demonstrate that this homozygous deletion causes subcortical heterotopic neurons, cytomegalic cells and rapamycin-sensitive aberrant mTORC1 signalling.^21^^,^^68^ As Old Order Mennonite patients with PMSE share the same deletion, they represent a homogeneous study population. Five patients with PMSE from the Old Order Mennonite community were treated with sirolimus before the onset of epilepsy (started at a mean age of 4.8 months). All patients had reduced seizures and improved receptive language compared with a cohort of historical controls.^21^ This early precision medicine trial provided a precedent for further study of mTOR inhibitor therapy for epilepsy and neurodevelopmental disorders in the mTORopathies.

### Tuberous sclerosis complex

TSC is the prototypical mTORopathy, characterized by multisystem benign tumours of the brain, skin, heart, lungs and kidney. Neuropathological findings include cortical and subcortical tubers, subependymal nodules and subependymal giant cell astrocytomas (SEGA).^8^ TSC is caused by inactivating *TSC1* or *TSC2* mutation, with germline pathogenic variants detected in over 80% of cases.^69^ Mosaic *TSC1* or *TSC2* variants were found in over half of cases lacking an identifiable germline mutation by conventional genetic testing.^70^*TSC1* encodes for hamartin and *TSC2* encodes for tuberin, both negative regulators of mTORC1 signalling.

Epilepsy is seen in 80–90% of cases that come to clinical attention.^71^ However, the exact incidence of epilepsy in TSC is unknown as many people with TSC without epilepsy will not seek medical attention. Nearly two-thirds of patients with epilepsy had seizure onset within the first year of life.^72^ Individuals may present with a variety of seizure types including focal onset seizures (with or without progression to bilateral tonic-clonic), epileptic spasms, and generalized onset seizures (tonic-clonic, atonic and atypical absence).^72^ DRE is common, occurring in two-thirds of patients with TSC, compared to one-third in the general epilepsy population.^72^ TSC-associated neuropsychiatric disorders (TAND) are a frequent occurrence in TSC, with intellectual difficulties and ASD occurring in approximately half of cases.^73^^,^^74^ Mental health issues occur in two-thirds of individuals with TSC, including depression, anxiety, attention deficit hyperactivity disorder (ADHD) and self-injurious behaviours.^75^*TSC2* pathogenic variants predict a more severe phenotype with a higher frequency of early-onset seizures, infantile spasms and developmental delay compared to patients with *TSC1* or mosaic *TSC2* variants.^74^^,^^76^^,^^77^ The more severe phenotype associated with *TSC2* variants may be explained by two factors. First, second-hit somatic *TSC1* variants appear to be less common than somatic *TSC2* variants.^78^ Indeed, cortical tuber counts are higher in *TSC2*-related disease which may be due to more frequent biallelic *TSC2* mutations.^69^ Second, loss of a single *TSC2* allele appears to have a more deleterious effect on the functional activity of the hamartin-tuberin complex compared with heterozygous *TSC1* variants.^50^

Historically, cortical tubers were considered the neuropathological substrate of epilepsy in TSC. However, perituberal tissues also display dysplastic neurons, giant cells, increased axonal connectivity and dysregulated mTORC1 signalling.^79^ Epilepsy surgery targeting removal of epileptogenic tubers and surrounding perituberal tissue is associated with better outcomes compared with resections that extend to the tuber margin only.^80^ Indeed, tuber-free mouse models of TSC exhibit increased expression of phosphorylated S6K and spontaneous seizures, suggesting that aberrant mTORC1 signalling alone may be sufficient to generate seizures.^49^ Epileptogenesis in TSC appears to be a progressive process. Cyst-like tubers are seen in almost half of individuals with TSC, and are associated with epileptic spasms and DRE.^81^ Serial MRI revealed that cyst-like tubers may increase in size and number. Moreover, they have a similar neuropathological appearance to some neurodegenerative white matter disorders, such as megalencephalic leukoencephalopathy with subcortical cysts.^81^ Interictal epileptiform discharges herald impending epilepsy in seizure-naïve infants with TSC.^82^ The EPISTOP study demonstrated that treatment with vigabatrin at the onset of epileptiform abnormalities on EEG delayed the onset of seizures, reduced the severity of epilepsy and reduced the frequency of neurodevelopmental delay compared with those who received vigabatrin after their first seizure.^83^

Dysregulated mTORC1 signalling results in the tumours, epilepsy and neuropsychiatric symptoms associated with TSC. Rapamycin and its analogues (or so-called ‘rapalogues’) are inhibitors of mTORC1 signalling and are established treatments for prophylaxis of organ rejection with kidney, liver and heart transplantation, and for cancers including renal cell carcinoma, breast cancer and pancreatic neuroendocrine tumours (Table 2).^84^ Rapalogues were developed to enhance the pharmacokinetic profile of their parent compound sirolimus. Rapamycin and its derivatives inhibit mTORC1 activity by an allosteric mechanism after forming a binding complex with FKBP12. Available mTOR inhibitors display limited penetration across intact blood–brain barrier, which may limit their efficacy in neurological disorders.^7^ In comparison to sirolimus, everolimus has superior pharmacokinetics and more robust clinical trial experience in oncology and TSC.^84^ Temsirolimus is a prodrug of sirolimus and is an approved intravenous treatment for advanced renal cell carcinoma.^85^ Ridaforolimus (also known as deforolimus) is an investigational mTOR inhibitor with some evidence for treatment of metastatic sarcoma.^86^

**Table 2:** Clinical pharmacology of rapamycin and its analogues (or rapalogues)

	Rapamycin	Everolimus	Temsirolimus	Ridaforolimus
	(sirolimus)			(deforolimus)
Biochemically functional form^84^	Sirolimus is active form	Active derivative (*hydroxyethyl ester)* of sirolimus	Prodrug of sirolimus (*activated after removal of dihydroxymethyl propionic acid ester*)	Active derivative (*dimethylphosphinate*) of sirolimus
Mode of administration	Oral, once daily	Oral, once daily	Intravenous, once weekly	Oral or intravenous
	Topical			
Protein binding^84^	∼92%	∼75%	∼85%	∼94%
Bioavailability^84^	∼15%	20%	100%	Tablet: 16%
Metabolism	CYP3A4,	CYP3A4, CYP3A5, CYP2C8	CYP3A4	CYP3A4,
	P-glycoprotein	(*hepatic metabolism 3-fold lower than sirolimus*)^85^		P-glycoprotein
Terminal half-life^85^	46–78 hours	26–30 hours	9–27 hours	30–75 hours
Elimination^85^	Faeces (91%),	Faeces (>90%),	Faeces (82%),	Faeces (88%),
	urine (2%)	urine (2%)	urine (5%)	urine (2%)
CNS penetration	Crosses BBB	Crosses BBB	Crosses BBB	Crosses BBB
	(*Preclinical data suggests poor CNS penetration*)^161^	(*Increased CNS penetration compared with sirolimus*)^161^	(*Decreased CNS penetration compared with sirolimus*)^162^	(*No data on CNS penetration compared with other agents*)
FKBP12 binding affinity	–	3-fold reduction in binding compared with sirolimus^163^	Similar binding affinity to sirolimus *(prodrug)*	3-fold reduction in binding compared with sirolimus^164^
Toxicity	Stomatitis: 42%^165^	Stomatitis: 25%^168^	Stomatitis: 27%^168^	Stomatitis: 54%^168^
	Pneumonitis: 1–14%^166^^,^^167^	Pneumonitis: 4–23%^90^^,^^167^^,^^169^^,^^170^	Pneumonitis: 2–6%^169^^,^^170^	Pneumonitis: 16%^86^
TSC-related indications	LAM	AML	Nil	Nil
	Facial angiofibromas	SEGA		
		Drug-resistant seizures		
Other indications	Immunosuppression in transplanted patients, PMSE	Immunosuppression in transplanted patients, kidney cancer, breast cancer and pancreatic tumours	Advanced kidney cancers	Investigational (sarcoma)

AML, angiomyolipoma; BBB, blood–brain barrier; CYP, cytochrome P450; FKBP12, FK506 binding protein 1 A 12 kDa; LAM, lymphangioleiomyomatosis; mTOR, mechanistic target of rapamycin; PMSE, polyhydramnios, megalencephaly and symptomatic epilepsy; SEGA, subependymal giant cell astrocytoma; TSC, tuberous sclerosis complex.

**Figure 2:**
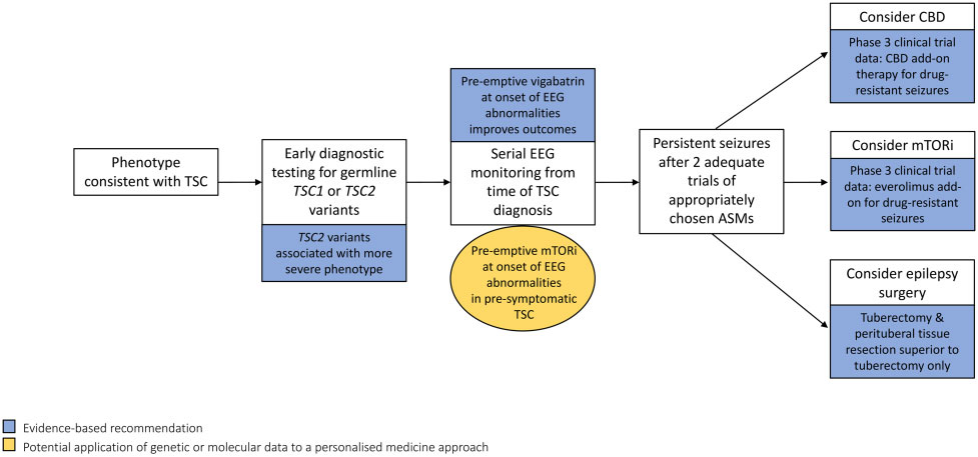
**A personalised medicine approach to the management of tuberous sclerosis complex-related epilepsy.** This figure outlines a therapeutic and prognostic framework, utilizing genetic and molecular data for the management of TSC-related epilepsy. Early genetic testing for *TSC1* or *TSC2* mutations is recommended for infants with phenotypic features of TSC. *TSC2* mutations are associated with a more severe neurological phenotype. In pre-symptomatic TSC, serial EEG monitoring is recommended, as pre-emptive vigabatrin at the onset of epileptiform abnormalities is associated with better long-term epilepsy outcomes. Evidence-based treatment options for TSC-related DRE include everolimus, CBD and tuberectomy with resection of surrounding perituberal tissue. Early treatment with everolimus in seizure-naïve TSC patients may improve long-term epilepsy and cognitive outcomes. Evidence-based recommendations are highlighted in blue and potential future applications are highlighted in gold. ASM, anti-seizure medication; CBD, cannabidiol; DRE, drug-resistant epilepsy; mTORi, mechanistic target of rapamycin inhibitor; TSC, tuberous sclerosis complex.

mTOR inhibitors are efficacious treatments for some of the tumours associated with TSC including sirolimus for pulmonary lymphangioleiomyomatosis (LAM) and renal angiomyolipoma (AML),^87^ everolimus for SEGA not amenable to surgery,^88^ and everolimus for AML.^89^ The EXIST-3 trial was the first large scale precision medicine trial for genetically mediated epilepsy. In this randomized, double-blind, placebo-controlled study, treatment with everolimus significantly reduced seizure frequency in individuals with TSC-related DRE. Forty percent of participants treated with high exposure everolimus (serum everolimus level of 9–15 ng/ml) had a greater than 50% reduction in seizure frequency compared with 15% treated with placebo.^12^ Increasing and sustained reductions in seizure frequency were observed in the EXIST-3 extension phase (Fig. 2).^90^

Everolimus demonstrated a favourable safety and tolerability profile in the EXIST-3 trial. Stomatitis was the most frequently reported complication, experienced to some degree by approximately 60% of participants. The incidence of adverse events decreased over time and complications rarely led to treatment discontinuation, with infrequent reports of serious infection and neutropenia.^12^^,^^90^ Real-world evidence from the TuberOus Sclerosis registry to increAse disease awareness (TOSCA) supports the safety and tolerability data from the EXIST trials. Over 60% of patients had an adverse event of any grade, of which stomatitis was the most common. Adverse events were manageable with dose reduction or temporary discontinuation, with a 95% retention rate over 5 years of observation.^91^ Similarly, in a survey-based study on the perspectives of patients with TSC treated with everolimus, adverse events were reported by 70% of participants. Overall tolerability was acceptable, with retention rates exceeding 80% after 3 years.^92^ Everolimus requires therapeutic drug monitoring due to its narrow therapeutic index and high inter- and intra-individual pharmacokinetic variability. Potent inducers of cytochrome P450 3A4, like carbamazepine and phenytoin, may lower the serum concentration of everolimus.^93^ Accordingly, dosing to a target everolimus level is challenging, particularly if blood draws are a source of distress for patients with comorbid intellectual difficulties and ASD. This challenge was apparent in the EXIST-3 trial, where many participants failed to achieve their target serum level, particularly in the high exposure group (target everolimus level 9–15 ng/ml).^12^

Protracted interruption of mTOR inhibitor treatment in TSC often leads to tumour regrowth or seizure worsening.^87^^,^^94^^,^^95^ Consequently, long-term treatment is recommended for TSC-related tumours and seizures. The long-term effects of mTOR inhibition in TSC are still being evaluated. Data from the extension phases of the EXIST trials do not indicate long-term effects on growth or sexual development.^88–90^^,^^94^ Real-world data from TOSCA found that patients treated with everolimus displayed age-appropriate sexual maturation.^91^ Metabolic complications, like hypercholesterolaemia and hypertriglyceridaemia occur in approximately 10% of patients treated with mTOR inhibitors, necessitating regular monitoring.^12^^,^^91^ Significant elevations in lipids or cholesterol can be managed by mTOR inhibitor dose reduction or anticholesterol agents. The long-term incidence of malignancy in solid organ transplant recipients taking mTOR inhibitors is low compared with those taking other immunosuppressive agents.^96^

Alternative mTOR inhibitor dosing regimens have been suggested in TSC that minimize drug exposure and side effects.^97^ Intermittent rapamycin dosing with ‘drug holidays’ maintained clinical efficacy in a mouse model of TSC.^98^ mTOR inhibitors display intrinsic and acquired treatment resistance in different human malignancies, and similar mechanisms may explain treatment failure in TSC. Incomplete inhibition of mTORC1 activity, failure to inhibit mTORC2 signalling, and mutations that disrupt FKBP12-rapamycin binding have been offered as potential mechanisms of resistance.^99^ Brain selective ATP-competitive mTOR kinase inhibitors that target mTORC1 and mTORC2 activity are being developed to overcome resistance mechanisms, improve CNS penetration and reduce systemic side effects.^100^ A recently published randomized control trial demonstrated that treatment with cannabidiol (CBD) reduced seizure frequency in TSC-related DRE. CBD has been shown to increase serum levels of everolimus and sirolimus. No patients in the CBD trial were taking concomitant mTOR inhibitors, so potential synergistic or toxic effects are unknown (Fig. 2).^101^^,^^102^

As pre-emptive vigabatrin improved epilepsy outcomes in TSC infants with abnormal EEG,^83^ additional benefits may be attainable with early everolimus therapy for seizure-naïve TSC patients. Indeed, early rapamycin prevented a severe neurological phenotype in rodent TSC models.^49^^,^^50^ In prospective cohorts of children and teenagers with TSC (age range 4–21 years), everolimus did not improve neurocognitive functioning, autism or neuropsychological deficits.^103^^,^^104^ A study of everolimus for the treatment of neurocognitive problems in TSC (TRON), involving patients aged 16–60 years is currently underway in the UK.^105^ Results from this placebo-controlled trial are not yet reported. However, it may be necessary to commence treatment earlier to observe a reduced incidence of TAND and DRE. A retrospective study of mTOR inhibitor treatment in patients with TSC under the age of 2 years (*n* = 17) found everolimus to be efficacious and safe for infants with cardiac rhabdomyoma, SEGA and epilepsy.^106^ However, larger prospective studies are needed to determine safety in this age category. In a mouse model of TSC, prenatal treatment with rapamycin led to hippocampus-dependent memory and learning deficits, which were not observed in mice treated postnatally.^107^ This suggests that early mTORC1 inhibition has the potential to alter pivotal anatomical structures involved in cognitive functions during neurodevelopment. An optimum ‘time window’ for early treatment, that maximizes epilepsy and cognitive outcomes without impacting neurodevelopment remains to be elucidated.

Prenatal diagnosis of TSC is possible with ultrasonographic detection of cardiac rhabdomyoma or SEGA. Neonates with TSC diagnosed prenatally and familial TSC cases could be targeted for recruitment in studies of mTOR inhibitors as prophylactic therapies for the manifestations of TSC. Kingsmore et al.^108^ demonstrated the value of early high-throughput sequencing in seriously ill infants with diseases of unknown aetiology. A genetic diagnosis was established in 23–43% of cases, of whom one-third had their treatment changed and one-fifth avoided major morbidity following an early molecular diagnosis.^108^^,^^109^ An early molecular TSC diagnosis would facilitate more accurate prognostication, especially in infants with pathogenic *TSC2* variants for whom a more severe phenotype can be predicted.^77^ Those with *TSC2* mutations could be prioritized for serial EEG monitoring and considered for early intervention with disease-modifying therapies. Ultimately, gene therapy may be a viable alternative to rapalogues, with the potential to improve long-term outcomes in TSC if administered early. In a mouse model of *TSC2* with prominent SEGA-like lesions, intravenous injection of an adeno-associated virus vector carrying a condensed form of tuberin led to reduced tumour volumes and improved survival.^110^

### The GATORopathies

GATOR1 functions as a negative regulator in the amino acid-sensing branch of the mTOR pathway. It is composed of three subunits: DEPDC5, NPRL2 and NPRL3. Heterozygous pathogenic variants in genes encoding the GATOR1 subcomplexes are a major cause of focal epilepsy and represent a distinct subset of mTORopathies, functionally subclassified as ‘GATORopathies’.^19^*DEPDC5* mutations were first identified as a common cause of familial focal epilepsy in 2013.^111^*NPRL2* and* NPRL3* variants were linked to focal epilepsy in 2016.^4^*DEPDC5* mutations account for 83% of all GATOR1-related epilepsies, while the remaining 17% is made up of *NPRL2* (6%) and *NPLR3* (11%) variants.^11^ The more recent discovery of epilepsy-causative *NPRL2* and* NPRL3* variants and the greater length of the *DEPDC5* transcript (5551 bp) compared with *NPRL2* (1700 bp) and *NPRL3* (2881 bp) have been offered as explanations for the increased frequency of *DEPDC5*-associated epilepsies.^11^

Pathogenic or likely pathogenic variants in the *DEPDC5, NPRL2* and* NPRL3* genes were identified in 8–11% of individuals within focal epilepsy cohorts, mostly comprised of familial non-lesional cases.^3^^,^^4^^,^^63^ The frequency of GATOR1-related epilepsies in these studies is likely over-estimates of their true prevalence due to the enrichment of familial cases in the cohorts. Large international collaborative studies have demonstrated the contribution of ultra-rare variation in known epilepsy genes to common epilepsies, like non-acquired focal epilepsy. In a study by the Epi4K and Epilepsy Phenome/Genome Project, approximately 3% of patients with familial non-lesional focal epilepsy had deleterious *DEPDC5* variants.^112^ The Epi25 Collaborative study found *DEPDC5* protein-truncating variants in 0.2% of patients with sporadic non-lesional focal epilepsy (Table 3).^113^

**Table 3:** A summary of the epidemiological, genetic and phenotypic characteristics of GATOR1-related epilepsies

Clinical characteristics	
Prevalence in focal epilepsy cohorts	
Focal epilepsy cohort (*n* = 404), mostly comprised of familial non-lesional cases^4^	9.4%
Focal epilepsy cohort (*n* = 93), mostly comprised of familial non-lesional cases^63^	11%
Non-lesional focal epilepsy cohort (*n* = 112, 66% sporadic)^3^	8%
Familial non-lesional focal epilepsy cohort (*n* = 525)^112^	2.6%
Sporadic non-lesional focal epilepsy cohort (*n* = 3400)^113^	0.2%
Distribution of GATOR1 variants^11^	
*DEPDC5*	83%
*NPLR2*	11%
*NPLR3*	6%
Mode of inheritance^11^	
*De novo*	4%
Inherited	96%
Frequency of mutation types^11^	
Loss-of-function	67%
Missense	27%
Splice-region	4%
In-frame deletion	1%
Penetrance^11^^,^^111^	66%
^a^Age of seizure onset^3^^,^^11^^,^^61–63^^,^^111^^,^^114–120^	mean= 9 years
	Range= 0–52 years
^b^Distribution of epilepsy phenotypes	
Nocturnal frontal lobe epilepsy^4^^,^^11^^,^^36^^,^^111^^,^^115–117^^,^^121^	42%
Temporal lobe epilepsy (including lateral)^3^^,^^4^^,^^11^^,^^111^^,^^118^	7%
Familial focal epilepsy with variable foci^3^^,^^36^^,^^63^^,^^111^^,^^114^^,^^115^	11%
^c^Other focal epilepsies^3^^,^^4^^,^^11^^,^^31^^,^^36^^,^^61–63^^,^^114^^,^^115^^,^^120^	26%
Epileptic spasms^11^^,^^119^	6%
Generalised epilepsy^11^^,^^31^^,^^127^^,^^145^	4%
Childhood epilepsy with centrotemporal spikes^120^	3%
Complex febrile seizures^11^	1%
^d^Frequency of malformations of cortical development^11^^,^^31^^,^^36^^,^^61–63^^,^^111^^,^^114–121^^,^^127^^,^^145^	23%
Frequency of drug-resistant epilepsy^11^	54%
^e^Frequency of sudden unexpected death in epilepsy in families with GATOR1 pathogenic variants^11^^,^^63^^,^^127^	9.3%
Frequency of cognitive comorbidities^11^	46%
Autism spectrum disorders^11^	9%
Frequency of psychiatric comorbidities^11^	43%
Oppositional disorder	18%
Attention deficit hyperactivity disorder	15%
Depression or anxiety	8%

a The mean age of seizure onset was calculated from a cohort of 268 individuals with GATOR1-related epilepsies reported in the literature.

b The distribution of epilepsy phenotypes was estimated from a collection of 152 GATOR1-related epilepsy pedigrees.

c Occipital lobe epilepsy, parietal lobe epilepsy or unspecified focal epilepsy.

d The frequency of malformations in GATOR1-related epilepsies was estimated from a collection of 143 pedigrees. Reported malformations of cortical development included focal cortical dysplasia type I and II, bottom-of-sulcus dysplasia. hemimegalencephaly, subcortical heterotopia, polymicrogyria and pachygyria.

e Fourteen SUDEP cases in 155 *DEPDC5* pedigrees; 1 SUDEP case in 10 *NPRL2* pedigrees; 2 SUDEP cases in 18 *NPRL3* pedigrees.^11^

The GATORopathies have a neurologic only phenotype, consisting of a broad spectrum of lesional and non-lesional epilepsies. The paradigmatic phenotype is familial focal epilepsy with variable foci (FFEVF).^4^^,^^11^^,^^111^^,^^114^^,^^115^ FFEVF is characterized by intrafamilial variability, with lesional and non-lesional epilepsies observed within families harbouring a particular GATOR1 variant.^20^ GATORopathies display incomplete penetrance, with variants dominantly inherited from asymptomatic parents in approximately 60% of cases.^11^ Germline GATOR1 variants have been identified in individuals and families with nocturnal frontal lobe epilepsy,^4^^,^^11^^,^^115–117^ temporal lobe epilepsy,^4^^,^^11^^,^^115^^,^^118^ epileptic spasms,^11^^,^^119^ and childhood epilepsy with centrotemporal spikes.^120^ Seizures occur predominantly from sleep in almost half of individuals with GATOR1-related epilepsies.^11^ Seizures usually begin in childhood or adolescence but the age of first seizure has ranged from the first days of life to older than 50 years.^3^^,^^11^^,^^63^^,^^111^ Importantly, over half of individuals with GATOR1-related epilepsies have DRE (Table 3).^11^

MCD were observed in over 20% of reported cases.^11^ FCD type II is the most common MCD seen in GATOR1-related epilepsies.^9–11^^,^^30^^,^^61^^,^^63^ FCD type I,^11^^,^^30^^,^^36^ BOSD,^26^^,^^61^^,^^121^ HME,^31^^,^^122^ polymicrogyria^4^ and subcortical band heterotopia^121^ have also been reported in individuals with GATORopathies (Table 3). Cognitive comorbidities are seen in approximately half of affected individuals and psychiatric disorders observed in over 40% of cases.^11^ Comorbid ASD has been described in approximately 9% of cases, with recent reports of an ASD only phenotype with *DEPDC5* variants.^11^^,^^123^ No distinct phenotypic characteristics have been identified when comparing individuals with *DEPDC5*, *NPRL2* or *NPRL3* variants, although FCD has rarely been reported with *NPRL2* mutations.^4^^,^^11^^,^^63^ The occurrence of second-hit somatic brain mutations may explain the wide interfamilial and intrafamilial phenotypic variability associated with germline GATOR1 subcomplex variants.^13^

Evidence is emerging for an increased risk of sudden unexpected death in epilepsy (SUDEP) with GATOR1 variants. In a series of 73 families harbouring GATOR1 subcomplex variants, nine individuals from eight families succumbed to definite or probable SUDEP. Definite SUDEP was confirmed by autopsy in one case, while the remaining eight cases had probable SUDEP (without autopsy confirmation). The mean age at the time of SUDEP was 36.8 years. Additional information, such as duration of epilepsy and years of observation was not available for some SUDEP cases.^11^ In a retrospective series of SUDEP cases (*n* = 61, 92% autopsy confirmed), 10% had bioinformatically inferred damaging *DEPDC5* variants.^124^ In preclinical models of GATOR1-related epilepsies, *Depdc5* conditional knockout mice display a propensity for terminal seizures, resembling the human phenomenon of SUDEP.^34^^,^^53^^,^^55^ Mice with focal cortical biallelic *Depdc5* deletions had clusters of focal onset tonic-clonic seizures, followed by EEG suppression, and in some cases death.^34^*Depdc5*, *Nprl2* and* Nprl3* expression analyses in mouse tissues revealed a general elevation in all brain regions compared with other organs, with expression highest in brain cortex.^4^ GATOR1 genes were expressed to a lesser degree in brainstem and heart tissues.^4^ Cardiovascular defects were seen in *Depdc5* and* Nprl3* mutant mice who died *in utero*, supporting roles for these genes in cardiovascular development.^125^^,^^126^ Further studies of rodent models may shed light on the cardiopulmonary and electrophysiological mechanisms underpinning SUDEP in GATOR1-related epilepsies. Drug resistance and nocturnal seizures are well recognized SUDEP risk factors, as well as being common features of GATORopathies. ‘Pseudoresistance’ may have contributed to SUDEP (autopsy-confirmed) in two brothers with the same *DEPDC5* pathogenic variant, as both were non-adherent with their anti-seizure medications (ASMs).^127^ It remains to be determined whether GATOR1 subcomplex mutations directly influence SUDEP risk or if the increased prevalence of SUDEP in GATOR1-related epilepsies merely reflects that it is a common cause of refractory focal epilepsy.

In a large cohort of individuals with epilepsy-related variants in GATOR1 genes, 96% were inherited and 4% occurred *de novo*.^11^ LoF variants account for 60–70% of the GATOR1 mutational spectrum and these largely consist of nonsense or frameshift variants (Table 3).^11^^,^^13^ Nonsense-mediated messenger RNA decay (NMD) was demonstrated in resected fresh-frozen brain,^61^ and cultured lymphoblasts^63^^,^^115^^,^^116^ obtained from individuals with *DEPDC5* and *NPRL3* nonsense variants, indicating that haploinsufficiency is the pathogenic mechanism leading to LoF and resulting loss of inhibition of mTORC1. Recurrent LoF variants have been reported, raising the possibility of mutational hotspots or founder effects.^11^ LoF variants predict a more severe phenotype, as they are associated with an increased frequency of FCD, epileptic spasms and SUDEP compared with missense variants.^11^ Missense variants were reported in over 30% of individuals with epilepsy-related variants in GATOR1 genes.^11^ Missense variants are more commonly associated with ‘MRI-negative’ non-lesional epilepsies.^13^*In vitro* functional assessments of GATOR1 missense variants have generally failed to demonstrate a deleterious effect on protein function.^57^^,^^58^ Moreover, strong evidence supporting their pathogenicity has not been obtained from familial segregation analyses or the presence of recurrent missense variants in unrelated probands. GATOR1 missense variants may result in an epilepsy phenotype through distinct effects on GATOR1 function or mTORC1-independent mechanisms.^11^ However, it is also possible that some GATOR1 missense variants previously predicted to be pathogenic, are non-contributory to the epilepsy phenotype, occurring in regions in the GATOR1 genes that are tolerant to variation. An adapted classification system has been proposed for clinical interpretation of GATOR1 missense and splice-region variants, using gnomAD allele frequencies and *in silico* pathogenicity predictions.^11^ It is unclear if this variant classification tool is ready for clinical application.

**Figure 3:**
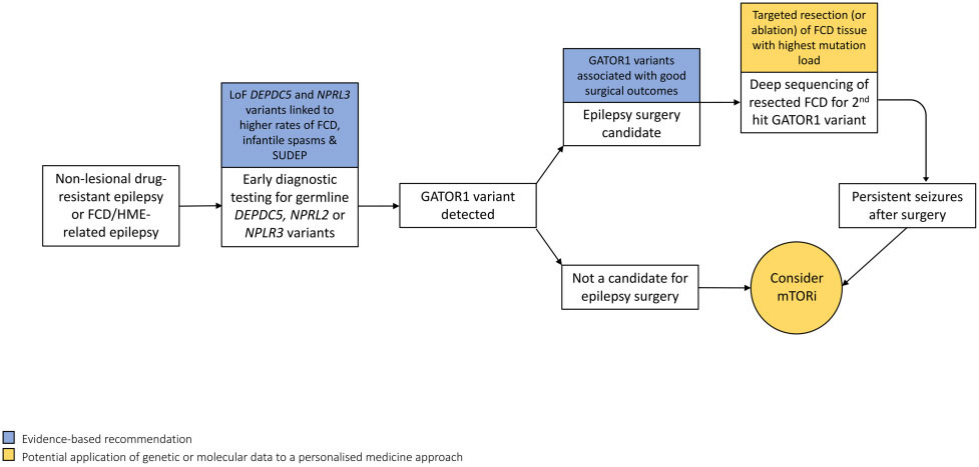
**A personalised medicine approach to the management of GATOR1-related epilepsies.** Early diagnostic sequencing of mTOR pathway genes is recommended in non-lesional epilepsies and in epilepsies due to FCD or HME. LoF *DEPDC5 and NPRL3* variants are associated with severe epilepsy phenotypes. Treatment options for GATOR1-related DRE include epilepsy surgery and potentially, mTOR inhibitors. Evidence-based recommendations are highlighted in blue and potential future applications are highlighted in gold. FCD, focal cortical dysplasia; GATOR1, GAP activity towards Rags 1 complex; HME, hemimegalencephaly; LoF, loss-of-function; mTORi, mechanistic target of rapamycin inhibitor; SUDEP, sudden unexpected death in epilepsy.

More efficacious therapies for GATOR1-related epilepsies are needed, as these disorders are frequently associated with DRE and a disproportionate risk of SUDEP.^11^^,^^124^ Excessive mTORC1 activation through dysregulated GATOR1 signalling appears to be involved in epileptogenesis in the GATORopathies. Preclinical rodent models,^52–55^*in vitro* functional studies^57^^,^^58^ and resected brain tissues^9^^,^^26^^,^^61–63^ from individuals with GATOR1-related epilepsies have demonstrated evidence of mTORC1 hyperactivation. As mTOR inhibitors have proven to be efficacious and safe treatments for DRE in TSC^12^ and PMSE,^21^ they represent a promising therapeutic strategy in GATOR1-related epilepsies. In a *Depdc5* conditional knockout mouse model, rapamycin reduced seizure frequency and extended survival.^55^ Treatment with sirolimus rendered an infant with *NPRL3*-related DRE seizure free after three weeks of therapy.^128^ Clinical studies are warranted to determine the effectiveness of mTOR inhibitors for DRE in the GATORopathies (Fig. 3). Individuals with GATOR1 inactivating nonsense variants may be targeted for mTOR inhibitor clinical trials, as there is proof for a LoF mechanism due to NMD.^61^^,^^63^^,^^115^^,^^116^ In contrast, individuals with missense variants may not be good candidates for treatment with mTOR inhibitors, as *in vitro* functional studies of missense mutations have failed to consistently demonstrate evidence of mTORC1 hyperactivation.^57^^,^^58^

Favourable epilepsy surgery outcomes have been reported in individuals with GATORopathies. In a cohort of patients with GATOR1-related epilepsies who underwent resective epilepsy surgery, 80% achieved a good surgery outcome (Engel score I–II).^11^ We recommend sequencing of GATOR1 genes during presurgical evaluations in those with non-lesional or FCD-related DRE for the following reasons: identification of a GATOR1 mutation may point to an occult underlying MCD in apparently non-lesional epilepsy; mTOR pathway mutations are associated with good surgical outcomes^129^; and mTOR inhibitors may offer an alternative therapeutic option, particularly in individuals with multifocal epilepsy, inoperable lesions or persistent seizures after surgery (Fig. 3).

### Focal cortical dysplasia and hemimegalencephaly

Somatic and germline mutations in genes encoding regulators of the mTOR signalling pathway are a major cause of FCD (including BOSD and HME). Individuals with FCD-related epilepsies account for 9% of the epilepsy surgery population.^130^ Brain somatic *MTOR* variants are the most common cause of FCD type II, accounting for approximately one-third of cases.^9^^,^^10^^,^^29–31^ Mosaic somatic variants in other mTOR pathway activators (*PI3KCA, AKT3, RHEB)* have been detected in FCD type II and HME resections.^9^^,^^10^^,^^27^^,^^29–31^^,^^60^ High read depth sequencing is required to improve diagnostic yield, as just under 80% of mutated FCD type II cases have brain mosaic rates lower than 5%.^131^ Sequence analysis of dysmorphic neurons and balloon cells extracted from FCD using single cell microdissection techniques may increase the rate of somatic mutation detection, as these neural cell subtypes are the main carriers of pathogenic variants.^9^ Germline inactivating *TSC1, TSC2, DEPDC5 and NPRL3* variants are present in patients with FCD type II and HME, with second-hit brain-confined somatic mutations reported in some cases.^9–11^^,^^30^^,^^31^^,^^33–35^^,^^61^^,^^63^

**Figure 4:**
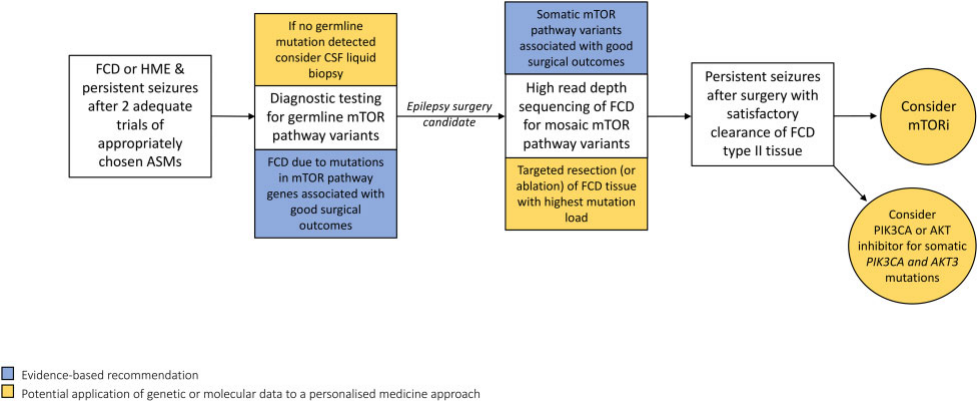
**A personalised medicine approach to the management of epilepsy due to FCD type II and hemimegalencephaly.** Epilepsy surgery for FCD type II and HME due to pathogenic variants in mTOR pathway genes is associated with good surgical outcomes. CSF liquid biopsy represents a less invasive option for identification of somatic mutations in cases without detectable mTOR pathway germline mutations. mTOR inhibitors may offer an alternative therapeutic strategy for individuals with inaccessible lesions or persistent seizures after surgery. PIK3CA and AKT inhibitors are promising targeted therapies for individuals with *PIKC3A-* and *AKT3-*associated MCD. Evidence-based recommendations are highlighted in blue and potential future applications are highlighted in gold. FCD, focal cortical dysplasia; HME, hemimegalencephaly; MCD, malformation of cortical development; mTORi, mechanistic target of rapamycin inhibitor.

Resective surgery for FCD caused by somatic or germline variants in mTOR regulatory genes is generally associated with favourable seizure outcomes (Engel I–II in 70–80% of cases).^9^^,^^11^^,^^129^ Diagnostic sequencing for pathogenic variants in mTOR pathway genes will become part of routine testing during presurgical evaluations for DRE caused by FCD or HME (Fig. 4). The mutational gradient observed in FCD type II tissue may have implications for surgical management.^9^^,^^26^ Within dysplastic specimens, the region with the highest rate of mosaicism correlates with the region of maximal epileptogenicity.^34^^,^^35^^,^^59^ Targeted resection or ablation of tissue with high mutational loads may be associated with improved surgical outcomes. Future FCD classification systems may include a molecular classification that will inform predictions of surgical outcomes and guide potential pharmacologic therapies.

The clinical utility of mTOR inhibitors for DRE caused by FCD type II remains to be determined. FCD type II surgical resections consistently display evidence of enhanced mTORC1 activation, even in specimens where low-level somatic mutations were not detectable.^9^ Rapamycin reduced seizures in rodent models of FCD,^28^^,^^51^ and in an infant awaiting hemispherectomy for HME (mosaic *MTOR* variant detected in resected tissue).^132^ mTOR inhibitors may be used as bridging therapy to reduce seizures in cases where immediate surgery is not possible. mTOR inhibitors may offer an alternative therapeutic strategy in individuals with surgically inaccessible FCD or for persistent seizures after epilepsy surgery (Fig. 4). A prospective, placebo-controlled clinical trial of everolimus is underway for patients with DRE due to FCD type II previously treated with surgery (NCT03198949).

Treatment with alpelisib (BYL719), a selective PIK3CA inhibitor improved clinical outcomes in individuals with *PIK3CA*-related overgrowth syndromes.^133^*PIK3CA*-related overgrowth syndromes are mosaic multisystem mTORopathies characterized by congenital lipomatous overgrowth, vascular malformations and skeletal abnormalities. Alpelisib is an oral treatment administered once daily. Some of the alpelisib-responders in the clinical trial had previously failed treatment with rapamycin.^133^ Somatic *PIK3CA* mutations have been detected in FCD type II, HME and polymicrogyria tissue.^9^^,^^10^^,^^23^^,^^27^^,^^30^^,^^31^ A child with HME in the context of *PIK3CA*-related overgrowth syndrome had reduced seizures and improved cognitive engagement at school following targeted treatment with miransertib (ARQ 092), an orally available AKT inhibitor.^134^ PIK3CA and AKT inhibitors are potential targeted therapies for MCD caused by somatic GoF mutations in *PIK3CA* or *AKT3* genes.

Analysis of brain tissue is a prerequisite for reliable detection of brain-confined somatic mutations using conventional sequencing approaches. In a large cohort study of children with FCD and HME, disease-causing somatic mutations were detected in brain resections using deep sequencing techniques in 46% of cases. Brain mosaic mutations were below the threshold for detection in 95% of paired blood samples.^9^ Less invasive methods that confirm the presence of somatic mosaicism are under development. Cell-free DNA is non-encapsulated, fragmented DNA found in bodily fluids, like blood and CSF. Liquid biopsy of cell-free DNA has emerged as an attractive alternative to tissue biopsy, with established applications in cancer diagnostics and disease monitoring. For example, sequence analysis of CSF-derived cell-free DNA is used to detect somatic mutations in patients with malignant brain tumours.^135^ In a recent study of CSF liquid biopsy for epilepsy-associated MCD, one-quarter of patients with somatic mutations previously detected in brain tissue (3/12), had the same mosaic variants detected in CSF-derived cell-free DNA. The mosaic pathogenic variants were absent from blood samples in all patients.^136^ Mosaic somatic mutations in the mTOR pathway genes *TSC1* and* PIK3CA* were detected in CSF-derived cell-free DNA obtained via dural puncture prior to surgery in two patients with FCD type IIB and HME, respectively.^136^^,^^137^ It remains to be seen if these findings can be replicated more broadly in CSF obtained from lumbar puncture. There are several potential clinical applications of CSF liquid biopsy in patients with FCD and HME. Establishing a genetic diagnosis prior to epilepsy surgery may assist prognostication. CSF liquid biopsy could be used as a surrogate for brain-derived DNA, facilitating early diagnosis of brain-confined somatic disorders and adoption of targeted therapies, such as everolimus for FCD caused by somatic mutations in mTOR pathway genes.

## Conclusions

In this review, we outlined the spectrum of mTORopathy-associated epilepsies, as well as highlighting the increased frequency of DRE in these disorders. As mTORC1 hyperactivation is a driver of epileptogenesis in the mTORopathies, rapalogues represent a more targeted therapeutic strategy compared with traditional ASMs. Everolimus has proven efficacy in TSC-related DRE,^12^ with rare reports of mTOR inhibitor use for epilepsy in non-TSC mTORopathies.^128^^,^^132^ Parallel group randomized controlled trials of mTOR inhibitors may not be feasible in rare mTORopathies, like the GATORopathies. N-of-1 studies are recommended for evaluating the efficacy of treatments in rare genetic disorders. N-of-1 studies are randomized, controlled, multiple crossover trials in single patients.^138^ Pooled data from multiple N-of-1 trials can produce robust treatment effect estimates.^139^ The N-of-1 study design offers an approach for providing evidence-based, personalized medicine. In addition, ‘N-of-some’ trials involving well organized clinical networks of patients with mutations in the same gene may facilitate precision therapy studies.

In genetic disorders with severe epilepsy phenotypes, there may be clinical justification to initiate off-label drug trials, particularly if the repurposed drug targets a genetic mechanism specific to the disorder. For example, two patients with *SCN8A*-related DRE experienced significant seizure reductions following off-label riluzole treatment. The decision to initiate riluzole was based on evidence from *in vitro* electrophysiological experiments, its favourable safety and tolerability profile in amyotrophic lateral sclerosis, and the severity of the patients’ epilepsy.^140^ Similarly, clinical justification for off-label treatment with everolimus in severe *DEPDC5*-related DRE is supported by favourable safety and tolerability data from TSC studies,^12^ and reports of rapamycin-responsive seizures in rodent models of *Depdc5* knockout.^55^ Explicit consent is a prerequisite when off-label treatments are used experimentally or as part of research. Obtaining informed consent from some patients with mTORopathy-associated epilepsies may be challenging, as intellectual disability is a common comorbidity. In select cases of severe GATOR1-related epilepsy, it may be in the patient’s best interest to initiate an off-label everolimus trial with the palliative goal of reducing seizures, after obtaining proxy assent from a family member or caregiver if the patient lacked decision-making capacity. Access to everolimus for TSC-related DRE is restricted in some jurisdictions. In the UK, everolimus candidacy is determined by a multidisciplinary team, which must include a neurologist with experience in TSC management and therapeutic drug monitoring. A multidisciplinary approach to decision-making on the use of repurposed treatments or novel precision therapies in rare genetic disorders will help facilitate appropriate patient selection for specific therapeutic interventions.

Personalized medicine strives to utilize an individual’s genetic and molecular data to inform clinical decisions and optimize therapies. Important prognostic information may be gathered from mTOR pathway-related genetic data including: *TSC2* variants are predicted to cause more severe neurological phenotypes in TSC^74^^,^^76^^,^^77^; and resective surgery for FCD caused by mutations in mTOR pathway genes is associated with good seizure outcomes.^9^^,^^11^^,^^129^ Here, we hypothesize that individuals with GATOR1 LoF variants linked to NMD may be more responsive to treatment with mTOR inhibitors compared with individuals with missense variants. For the reasons outlined above, we anticipate that diagnostic sequencing for mTOR pathway genes will become routine practice in the assessment of individuals with DRE. With gene therapies on the horizon, establishing a high-definition molecular genetic diagnosis is more important than ever.

## Funding

This publication has emanated from research supported in part by a research grant from Science Foundation Ireland under Grant Number 16/RC/3948 and co-funded under the European Regional Development Fund and by FutureNeuro industry partners. P.B.M. receives funding from the Royal College of Surgeons in Ireland—Blackrock Clinic StAR MD programme, 2020. N.D. has received an unrestricted grant from Novartis Ireland to support the establishment of an epilepsy genetics registry.

## Competing interests

The authors report no competing interests.

## Data availability

This review article did not involve the generation of new data.

## Abbreviations

AML =: angiomyolipoma
ASD =: autism spectrum disorder
ASM =: anti-seizure medication
BOSD =: bottom-of-sulcus dysplasia
CBD =: cannabidiol
DEE =: developmental and epileptic encephalopathy
DEPDC5 =: Dishevelled, Egl-10, and Pleckstrin domain-containing protein 5
DRE =: drug-resistant epilepsy
FCD =: focal cortical dysplasia
FFEVF =: familial focal epilepsy with variable foci
FKBP12 =: FK506 binding protein 1 A 12 kDa
GAP =: GTPase-activating protein
GATOR1 =: GAP activity towards Rags 1 complex
GoF =: gain-of-function
HME =: hemimegalencephaly
LAM =: lymphangioleiomyomatosis
LoF =: loss-of-function
MCD =: malformation of cortical development
mTOR =: mechanistic target of rapamycin
mTORC1 =: mechanistic target of rapamycin complex 1
NMD =: nonsense-mediated messenger RNA decay
NPRL2 =: nitrogen permease regulator-like 2
NPRL3 =: nitrogen permease regulator-like 3
PI3K =: phosphoinositide 3-kinase
PMSE =: polyhydramnios, megalencephaly and symptomatic epilepsy
PTEN =: phosphatase and tensin homologue
Rheb =: Ras homologue enriched in brain
SEGA =: subependymal giant cell astrocytoma
STRADα =: STE20-related kinase adaptor alpha
SUDEP =: sudden unexpected death in epilepsy
S6K =: ribosomal S6 kinase
TAND =: tuberous sclerosis complex-associated neuropsychiatric disorders
TSC =: tuberous sclerosis complex
